# Chemical-Saving Potential for Membrane Bioreactor (MBR) Processes Based on Long-Term Pilot Trials

**DOI:** 10.3390/membranes14060126

**Published:** 2024-05-29

**Authors:** Sofia Lovisa Andersson, Christian Baresel, Sofia Andersson, Klara Westling, Mikael Eriksson, Andrea Carranza Munoz, Gabriel Persson, Mayumi Narongin-Fujikawa, Kristin Johansson, Tomas Rydberg

**Affiliations:** 1 Stockholm Vatten Och Avfall AB, 106 36 Stockholm, Sweden; 2 IVL Swedish Environmental Research Institute, P.O. Box 21060, 100 31 Stockholm, Swedentomas.rydberg@ivl.se (T.R.); 3 Sweco Environment, Gjörwellsgatan 22, 112 60 Stockholm, Sweden; 4 Svenskt Vatten, P.O. Box 14057, 167 14 Bromma, Sweden

**Keywords:** wastewater treatment, membrane bioreactor, membrane cleaning, cleaning chemicals, resource efficiency, environmental impact, cost saving

## Abstract

Membrane bioreactors (MBRs) have gained attraction in municipal wastewater treatment because of their capacity to meet strict water quality standards and support water reuse. Despite this, their operational sustainability is often compromised by high resource consumption, especially regarding the use of chemicals for membrane cleaning. This study explores innovative membrane-cleaning strategies to enhance the sustainability of MBR processes. Through long-term pilot trials at Stockholm’s largest wastewater treatment plant, this study showed that alternative cleaning strategies can reduce chemical use by up to 75% without sacrificing treatment performance. The results further suggest that these alternative strategies could result in cost reductions of up to 70% and a reduction in environmental impacts by as much as 95% for certain indicators. Given that MBRs play a crucial role in addressing increasing treatment demands and advancing circular water management, the outcomes of this study are beneficial for the broader adoption of MBR processes. These results also have implications for existing installations, offering a pathway to more sustainable wastewater treatment. Moreover, the presented cleaning strategies provide significant opportunities for lowering operational costs and reducing the environmental footprint of new and existing MBR installations.

## 1. Introduction

Membrane bioreactors (MBRs) are gaining increasing popularity in municipal wastewater treatment with an exponential increase in the number and scale of MBR facilities for municipal wastewater treatment [1,2,3,4]. The driving factors are often of multiple nature including increased loads due to urbanization, limited space for expansion in urban wastewater treatment plants (WWTPs), and more stringent treatment requirements to avoid pollution of the aquatic environment. Also, the increasing demand for the implementation of water reuse schemes based on municipal wastewater implies increasing interest in the MBR system, e.g. [5,6]. This is thanks to MBRs low footprint, robust operation, and the possibility to meet high-quality demands on the treated water.

A key disadvantage often associated with MBR processes is their higher resource consumption, particularly in terms of chemicals and energy, compared with conventional activated sludge (CAS) systems. This issue is mainly linked to membrane cleaning during operation to prevent fouling, which involves significant energy use for physical cleaning (membrane aeration) and substantial chemical use in both maintenance and recovery cleanings. Additionally, the need to replace the membranes after a few years represents a significant operational cost. Membrane aging, which drives the need for replacement, is strongly influenced by chemical cleaning, as the cleaning process is the primary cause of membrane degradation and disintegration [7,8].

On the other hand, chemical cleaning is an inevitable part of the MBR process, especially when the discharge limits of phosphorus are low, as an increase in precipitation chemical consumption will result in increased membrane fouling [9]. Sodium hypochlorite (NaOCl) and citric acid (C_6_H_8_O_7_) remain the most common chemicals used for membrane cleaning [10]. Besides cost and environmental impacts related to the manufacturing of chemicals, sodium hypochlorite is associated with negative impacts such as the formation of toxic gases and byproducts [11]. For flat sheet membranes, oxalic acid ((COOH)_2_) has also been used for membrane cleaning but is usually not utilized for cleaning hollow fiber membranes.

The overall sustainability of wastewater treatment is becoming increasingly important in society’s efforts to balance environmental impacts and living standards, and in light of the adaption to global challenges such as climate impact and biodiversity loss. The fact that MBR systems can achieve a much higher effluent quality than CAS systems is not, by itself, a sufficient justification for preferring MBR over CAS processes. To make MBR processes more sustainable, it is crucial to reduce resource consumption during operation and extend the life span of the membranes, thereby conserving resources and lowering operational costs.

Various suggestions to reduce the environmental impact of MBR processes, especially related to membrane fouling, have been suggested in the literature in recent years. These ideas include novel membrane materials to reduce fouling, e.g. [12,13,14], biological control strategies, e.g. [15,16], and using alternative cleaning agents [17] or other physical approaches such as, e.g. movable membranes [18], the addition of particles for physical scouring, e.g. [19,20], or hybrid electrochemical approaches, e.g. [21]. Although some of these suggestions have the potential to improve resource efficiency and thus minimize the environmental impact of MBR processes, they generally represent approaches in an early research stage requiring years of research, development, and verification before their eventual acceptance for implementation in full-scale MBR processes.

The current work aims to provide recommendations for optimizing municipal MBR processes instantly to increase resource efficiency based on long-term pilot tests. The experimental trials are performed in preparation for one of the world’s largest municipal MBR facilities that will be started in Stockholm, Sweden, and thus are directly relevant for full-scale implementation. Reduced chemical consumption during membrane cleaning while maintaining functionality would not only reduce the negative environmental impacts from production, transportation, and use of these chemicals, it would further prolong the membrane lifetime and reduce negative impacts originating from, e.g., chlorinated gas emission and the formation of chlorinated compounds such as adsorbable and extractable organic halogens (AOX and EOX). Reduced cleaning intervals would generally also reduce the downtime of the membranes and thus increase the overall capacity and recovery rate of MBR processes.

The presented work focuses on chemical consumption and the related reduction in their use for fouling control without losing membrane functionality in existing MBR processes. As such, a broad implementation of these findings could be accomplished.

## 2. Materials and Methods

### 2.1. Henriksdal WWTP, Stockholm

Stockholm is growing rapidly, which suggests an increased load on WWTPs. At the same time, more stringent effluent quality demands (biochemical oxygen demand, 7 days: 5 mg BOD_7_/L, total nitrogen: 6 mg TN/L, and total phosphorus: 0.20 mg TP/L, plus an annual TP quantity requirement) are being implemented. Stockholm Water and Waste Company (Stockholm Vatten och Avfall AB, Stockholm, Sweden) has two large WWTPs, Bromma (300,000 pe (population equivalent, defined as 70 g BOD_7_ per person and day)) and Henriksdal (950,000 pe). Both plants require extensive rehabilitation, are operated well above their design capacity, and have no space for expansion. The selected solution is to close down Bromma WWTP (~2026), build a new tunnel (ongoing), and upgrade Henriksdal WWTP to a capacity of 1.6 Mpe with MBR technology (ongoing) without extending biological treatment volumes. The future Henriksdal WWTP will be Europe’s largest municipal MBR process.

The future Henriksdal MBR process includes a pre- and post-denitrification process using methanol as an external carbon source for nitrogen removal. For phosphorus removal, the design includes both pre- and simultaneous precipitation using a combination of ferrous and ferric products dosed at three points along the process. The first of seven treatment lines was commissioned in January 2021, and after almost one year of trimming, it was put into normal operation in September 2021.

Henriksdal membranes were designed to be cleaned using citric acid for inorganic (iron-based) fouling and sodium hypochlorite to remove organic fouling. However, preparations capable of switching to oxalic acid were made from the start based on results from initial pilot trials. Citric acid was also used from start-up in January 2021 to November 2023 when citric acid was replaced with oxalic acid based on the results presented in this work and the fact that oxalic acid is gentler on the concrete in the basins compared with citric acid. The design includes two types of cleaning procedures including maintenance cleaning (MC, lasts ~1 h), carried out as a chemically enhanced backwash (CEB) two times per week using sodium hypochlorite and once per week using citric acid, and recovery cleaning (RC, membranes soaked in chemical solution for 6–16 h), carried out twice per year with sodium hypochlorite and twice per year using citric acid.

### 2.2. Pilot Characteristics

**Figure 1:**
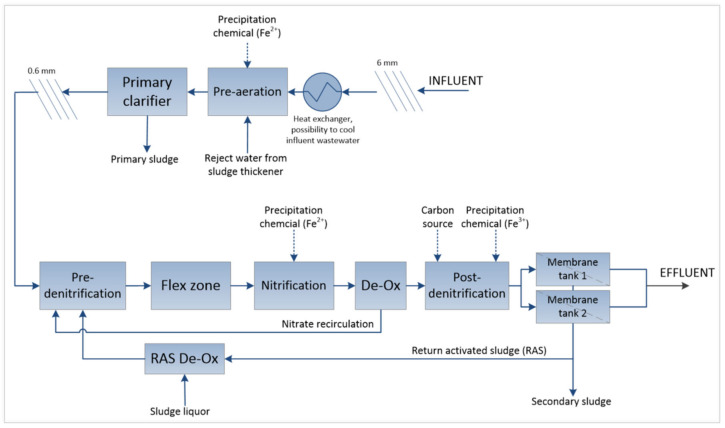
MBR pilot process configuration (sludge treatment excluded in the figure).

In order to verify, develop, and optimize the process design, pilot trials (scale 1:6500) have been carried out at the R&D facility, Hammarby Sjöstadsverk, Stockholm, Sweden, since 2013, with a pilot including water and sludge treatment. The pilot has been continuously operated with municipal wastewater from the city of Stockholm flowing to the current Henriksdal WWTP, Stockholm, Sweden. The pilot was mainly operated with dynamic inflow (average flow up to 4.4 m^3^/h) linked to the actual inflow measurement at the Henriksdal WWTP. The sludge concentration in the biological treatment was on average 7600 mg SS (suspended solids)/L, with a sludge age of 17.5 d and specific air flow for membrane operation of 0.136 and 0.252 Nm^3^/h, m^2^ for the two operating modes Leap-low and Leap-high, respectively. Since 2016, the membranes used in the pilot have consisted of Zeeweed 500D (Veolia) ultrafiltration (UF) hollow fiber membranes (nominal pore size of 0.04 μm) installed in two parallel membrane tanks. This corresponds to the same membrane units used in the full-scale Henriksdal WWTP. The pilot process configuration is presented in Figure 1.

The standard operational procedure for the membranes was cycles of 10 min filtration and 1 min relaxation (membrane scouring air was on during the entire cycle). The net flux varied between 18 and 30 L/(m^2^·h) (liters of permeate per square meter of membrane area per hour) to handle the dynamic influent. During the years of pilot operation, many tests have been carried out, for example, different cycle times, backwash instead of relaxation, different flux settings, and varying sludge concentrations. However, in this paper, only the main chemical cleaning procedures tested on Zeeweed 500D membranes (Veolia) are presented.

### 2.3. Pilot Membrane Operation and Cleaning

The two parallel pilot membrane tanks (MT1 and MT2) were operated in an identical way with the following exception: MT1 was cleaned using oxalic acid, while MT2 was cleaned with citric acid. The different acids were used for both maintenance cleaning (MC) and recovery cleaning (RC). While MC is a regular light cleaning of the membranes to keep performance steady, RC is a more intense cleaning, which soaks the complete membrane in cleaning chemicals to fix significant fouling and restore performance. The reason for choosing oxalic acid for tests in this study was mainly because (1) it is a common chemical used for flat sheet membranes that were tested in the pilot before and (2) the membrane supplier accepted this to be used and provided a design cleaning scheme for this chemical. However, to the authors’ best knowledge, oxalic acid has not previously been tested on municipal MBRs with ZW500D membranes. Oxalic acid is also cheaper than citric acid. Another observed environmental advantage of oxalic acid is that it also prevents phosphorous peaks following cleaning with citric acid, representing a challenge for the annual TP quantity requirement [22]. Thus, oxalic acids can be a better choice for MBR installations using ferrous as a precipitation agent. The main drawback of using oxalic acid on a full scale is the space and equipment required to store larger volumes of chemicals (oxalic acid is only 8% solution while citric acid is 50%). In addition, citric acid can be more aggressive to concrete constructions compared with oxalic acid [23].

The membrane suppliers’ recommended cleaning procedures are summarized in Table 1. Sodium hypochlorite was delivered as a 10–20% solution, citric acid was delivered as a 51% solution, and oxalic acid was delivered in powder form and mixed with water to saturation (resulting in an 8% solution). The cleaning chemicals were diluted with permeate to the concentrations specified in Table 1 during MC and RC.

The membranes were initially cleaned according to the recommendations (Table 1), and the evaluation focused on comparing citric and oxalic acid. When the optimization period started, the MC cleaning frequency and chemical amount added per cleaning (either by reduced concentration in the CEB or by reduced time for the CEB) were applied for MT1 and MT2. All other operational settings were the same for the two tanks. The evaluation was divided into test periods with different settings for the cleaning procedures (also see Table S1 in the Supporting Information for more details). Here, the test periods were aggregated into three groups following the order of the optimization procedure as follows:

1. Oxalic acid MC
  - Stepwise, the frequency, the number of CEBs, and CEB concentration were minimized while maintaining permeability similar to the reference line and always above 200 L/(m^2^·h·bar).
2. Citric acid MC
  - Starting at optimal settings for oxalic acid MC obtained in step 1, and then adjusting to ensure no negative impact on permeability.
3. Sodium hypochlorite MC
  - Operating without adding any sodium hypochlorite until permeability decreased below 200 L/(m^2^·h·bar).
  - Introducing demand-driven MC by using membrane resistance and an algorithm that automatically decided if cleaning was to start.

Initially, optimization was only made for oxalic acid (as this had not been tested before). MT2 was kept as a reference with citric acid design cleaning while the cleaning frequency, CEB time, and concentration for MT2 with oxalic acid were reduced in steps. To ensure a fair comparison of the two acids, an optimization of the citric MC followed. Finally, the sodium hypochlorite MC was optimized for both membrane tanks (Table S1, Supporting Information).

Recovery cleanings were carried out according to recommendations (once or twice per year) during the complete trial.

Two operation modes with regard to the membrane cleaning strategy can be defined based on the long-term trials in the pilot. The “optimized design operation mode” is defined as the reduced frequencies possible to implement at full scale, and the “demand-driven operation mode” is defined as a mode when cleaning events are triggered by an algorithm using membrane resistance during the filtration cycle to decide if cleaning is needed.

The “optimized design operation mode” was still time-based, while the “demand-driven operation mode” frequency of MCs was decided by monitoring membrane resistance. If the membrane resistance passed a threshold value, this called for cleaning, and an MC with sodium hypochlorite was carried out. Every 4th MC with sodium hypochlorite was also complemented with an acid MC. The number of CEBs for sodium hypochlorite MC was reduced from 8 to 6 during the entire trial.

For other operational and pilot-specific information, please see [24].

### 2.4. Environmental Impact Assessment

Data for the environmental impact of citric acid and sodium hypochlorite for the different impact categories were collected from the databases Thinkstep [25] and Ecoinvent [26]. For oxalic acid, no data were registered in the Ecoinvent environmental database when the impact assessment was initiated. In addition, no scientific publications on the environmental impacts of oxalic acid were found, and production-related data were not provided by the contacted suppliers (e.g., Brenntag, Wibax). There are several routes in place to produce oxalic acid commercially, and these may differ significantly in the use of resources, e.g. [27]. In 2022, data for oxalic acid production became available in the Ecoinvent database (version 3.8) but for a production process that can be considered least favorable regarding environmental impacts.

To illustrate the difference in environmental performance for two significantly different production routes, both a process route based on a carbohydrate-rich substrate of biogenic origin (“biobased production”) and an alternative route based on the oxidation of fossil propylene (“fossil-based production”), as available in the commercial database eco-invent, were considered in the impact assessment. For “sustainable production”, a simple Life Cycle Assessment (LCA) model was established in the software GaBi, version 9.2.1 [25].

“Biogenic production” (simplified LCA model): A large share of the global market of industrial oxalic acid is made available through the biogenic route [27]. The process is based on the oxidation of carbohydrates to oxalic acid by means of nitric acid. The raw precipitate of oxalic acid is then recrystallized to higher purity. Various production processes have been described in the literature, and the model created here is based on a process design by [28]. The overall reaction formula is

C_6_H_12_O_6_ + 6HNO_3_ ⇨ 3[COOH]_2_·6H_2_O + 6NO

Thus, 3 moles of oxalic acid are theoretically produced from one mole of hexose, requiring 6 moles of nitric acid. Translated to mass units, 126 kg of nitric acid is required for 90 kg of oxalic acid, or 1.4 kg of nitric acid per kg of oxalic acid. In subsequent reactions, nitric oxide (NO) is further reacted in two steps by oxidation to nitrogen dioxide (NO_2_) and then absorbed in an aqueous medium back to equal mole fractions of nitric acid and nitric oxide. The resulting nitric acid outflow from the regeneration process cannot be directly reused because it has a lower concentration. No credit is therefore assigned to the HNO_3_ output. LCA data for nitric acid and oxygen were also taken from the Thinkstep and Ecoinvent databases, and the impact calculation was based on the following assumptions:

- The carbo-hydrate raw material is a waste or by-product from other processes and, as such, carries no burden from upstream processes.
- The raw material requirements for the process are 1.4 kg of nitric acid and 0.32 kg of oxygen.
- As the oxalic acid reaction is strongly exothermal, the heat of the formed reaction balances the heating needed for other process steps.
- Various electricity requirements for pumping, compressing, etc., are lumped into an overall electricity need for 1 kWh/kg oxalic acid.
- A fugitive loss of 14 g NO/kg oxalic acid is assumed to be emitted into the atmosphere, corresponding to 98% capturing efficiency in the scrubber where nitric acid is regenerated.

The production location of the delivered oxalic acid was assumed to be Spain, India, or China, and the LCA model was applied for these different locations in the sense of the used energy mix (see Table S2, Supporting Information). Transport of the product to Sweden was not included but is considered a minor aspect compared with the production process.

“Fossil-based production” (Ecoinvent data): The fossil-based route represents the production of 1 kg of oxalic acid from propylene, as modeled by [29], based on a two-step method developed by Rhône-Poulenc. In this process, in the first step, propylene is partially oxidized to alpha-nitrolactic acid, which, in the second step, is further oxidized with oxygen to oxalic acid.

### 2.5. Cost Calculations

For cost calculations, actual prices for chemicals used in the full-scale MBR process at Henriksdal WWTP were derived. Even if these prices represent the current situation at Henriksdal WWTP, there has been a general increase in prices for process chemicals because of an energy crisis in Europe. This implies that savings in actual money may change in the future when considering increased prices. Only comparative cost calculations were performed to obtain cost savings. As such, no life cycle cost or other more holistic cost analyses were performed.

## 3. Results and Discussion

### 3.1. General Pilot Performance

Based on six years of pilot operation, it can be concluded that the future discharge limits at Henriksdal WWTP can be achieved. Specifically, regarding nitrogen (TN and ammonium NH_4_-N) and BOD_7_, the yearly effluent average was well below the expected limit from the start, as shown in Table 2. Since the membrane tank (MT) is continuously aerated, any possible ammonium entering from the biological treatment step will be nitrified; therefore, the ammonia levels in the effluent were close to 0 mg/L throughout the pilot operation. Regarding TP, because of the risk of fouling the membranes, the dosing of the precipitation chemical was initially restrictive, and then slowly increased, resulting in lower effluent TP-values from 2017 onward (and a precipitation chemical consumption of 20–30 mg Fe/L). In the following years, spontaneous biological phosphorus removal occurred in the pilot, although it was not designed for enhanced biological phosphorus removal (EBPR). This contributed to a lower yearly consumption of precipitation chemicals and low effluent total phosphorus concentrations.

### 3.2. Permeability and Consumption of Membrane Cleaning Chemicals

**Figure 2:**
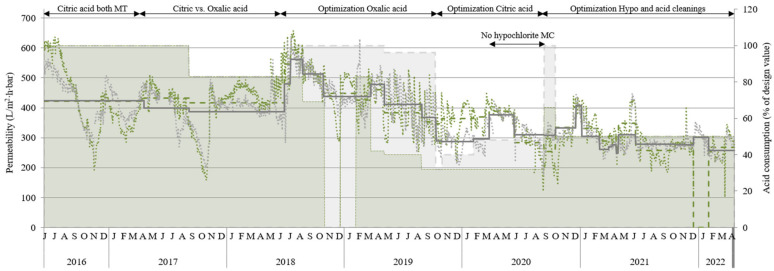
Permeability for MT1 (oxalic) and MT2 (citric) as daily averages and as averages for each trial. Also shown in the graph is the amount of acid used for MC cleaning compared to design cleaning (100%).

Figure 2 shows the permeability during the past project years including various operation modes for membrane cleaning. The cleaning optimization test was aggregated into four main groups as follows (see also Table S1, Supporting Information):

1. Design cleaning (June 2016 to June 2018).
2. Optimization of oxalic acid MC (June 2018 to October 2019).
3. Optimization of citric acid MC (October 2019 to February 2020).
4. Optimization of sodium hypochlorite MC in combination with acid MC (February 2020 to April 2022).

Permeability was similar or slightly higher when using oxalic acid (MT1) compared with citric acid (MT2). Oxalic acid consumption could be lowered even more than citric acid (compared with design consumption) while still maintaining performance; however, both could be reduced by 50% while maintaining good permeability (see permeability and chemical consumption from September 2019 onward in Figure 2). When sodium hypochlorite and acid cleanings were synchronized (carried out the same day), a more distinct effect of the maintenance cleaning was observed, causing the permeability to vary more (see 2019); however, on average, the permeability was about the same as when cleanings were carried out non-synchronized (different days of the week). More information about the various optimization trials can also be found in Table S1 in the Supporting Information.

Generally, the membranes performed well with reduced chemical cleaning; therefore, some drastic tests were performed. In October 2018, it was decided to stop the oxalic acid cleaning for MT1 completely and await a permeability decrease. After 53 days of operation, permeability decreased from 544 to 289 L/(m^2^·h·bar). One maintenance cleaning with oxalic acid restored the permeability almost completely to 507 L/(m^2^·h·bar). MT1 continued to operate without any oxalic acid for another 49 days before this trial was stopped.

In March 2020, sodium hypochlorite was replaced with water to see the effect of the back pulse alone without the addition of any chemicals. As permeability remained stable for more than two months with this approach, it was tested to exclude the sodium hypochlorite MCs completely and monitor how long the membranes could operate with only acid MCs before permeability decreased below 150 L/(m^2^·h·bar). This trial started in June and continued for three months. The membranes were operated without sodium hypochlorite MC for 92 days, and permeability decreased from a maximum of 394 L/(m^2^·h·bar) to a minimum of 121 L/(m^2^·h·bar) for MT1 and from 383 to 175 L/(m^2^·h·bar) for MT2. This resulted in a slow reduction in permeability followed by more sudden drops in permeability (especially for MT1), but permeability was only below 200 L/(m^2^·h·bar) for 9 days (MT1) and 1 day (MT2) and was easily recovered by initiating standard cleaning.

Although these two tests suggest that the membranes can be managed for long periods with much less chemical cleaning compared with design cleaning, there is a balance with physical membrane cleaning using scouring air. To not risk the need for higher aeration resulting from reduced chemical cleaning, a reasonable middle-way was reached by using the control algorithm for membrane aeration (which is based on membrane resistance) to trigger sodium hypochlorite MC instead of initiating higher aeration. The acid MCs were carried out according to design settings but with double intervals (once every other week instead of once per week), which corresponds to half consumption. This demand-driven mode was tested from October 2020 to the end of the project in April 2022. During this period several other tests with negative effects on membrane permeability (high flux, limited membrane aeration, and reduced recirculation flowrate) were carried out in parallel. Even so, if looking at the entire period, the average frequency for sodium hypochlorite MC was more than 4 times less compared with design cleaning. The sodium hypochlorite cleaning was on average carried out after permeate production of 763 m^3^ and 625 m^3^ for MT1 and MT2, respectively, compared with design cleaning after 179 m^3^. December to February was excluded because MT2 was out of operation during this period. If looking at specific cleanings, some were carried out after more than 2900 m^3^, which corresponds to about 6% of the design cleaning.

The chemical consumption in the MBR pilot and the Henriksdal full-scale design is summarized in Table 3. The design consumption is compared to the two cases of operational modes including the following:

1. Optimized design operation mode—This mode represents an optimization level that can be currently implemented on the full scale. This operational mode means a 50% reduction in chemical consumption for RC (1 RC/year instead of 2) and a 20% reduction for MC.
2. Demand-driven operational mode—This case represents the best total optimization achieved with stable results in the pilot without considering the membrane warranty.

Generally, the consumption of cleaning chemicals in the pilot was considerably lower than the design values. In total, the amount of chemicals used for each RC was the same as in the design; however, in most years, RC was only required once, while it is designed for use twice per year. In addition, the pilot inflow was higher than the design flow.

The data presented in the demand-driven operation mode in Table 3 are based on RC once per year and a flowrate 12% higher than the design flow, resulting in a chemical consumption corresponding to 44% of the design.

For MC, the demand-driven operation mode for citric acid was the same as the design (eight back pulses with design concentration); for oxalic acid, it was six back pulses instead of eight and 80% of the design concentration in the CEB. For sodium hypochlorite, it was six back pulses and the design concentration. The frequency for sodium hypochlorite was reduced to 29% and 38% of the design frequency for MT1 and MT2, respectively (one cleaning per 627 m^3^ and 468 m^3^ of treated water on average), and the acid MCs were performed every other week instead of once per week.

The acid chemical consumption for MC, however, was only 32% of the pilot design values for oxalic acid and 48% for citric acid. MT1 (which was cleaned with oxalic acid) required fewer sodium hypochlorite cleanings compared with MT2. During the more than a year-long trial (including tough operating conditions for the membranes), the average sodium hypochlorite consumption for MC corresponded to 24% and 34% of the design consumption for MT1 and MT2, respectively. In Table 3 and future calculations, 24% was used, partly because it was the best result based on more than one year of operation. MT2 also performed better during some of the trials, and it was considered reasonable for MT2 to achieve this level when the membrane operation was in normal conditions.

The design values are based on the pilot design inflow of 3.2 m^3^/h, while a much higher actual inflow was considered in the pilot values. The results indicate that costs and environmental impact can be significantly reduced in the full-scale plant by reducing the consumption of cleaning chemicals. Even though the same MC intervals as in the design are applied, the number and timing of back pulses, amount of chemicals, etc., were optimized. Also, the cleaning interval was investigated with demand-driven cleaning instead of fixed intervals.

### 3.3. Environment Impact

The environmental impact of the different acids for membrane cleaning is provided in Table 4 for the major impact categories. It can be observed that oxalic acid has a lower or higher environmental impact per produced kg than citric acid depending on which production process is used. Based on the simplified LCA model for sustainable production of oxalic acid, the global warming potential (GWP) and abiotic depletion potential (ADP) are about 30% lower, the acidification potential (AP) and photochemical ozone creation potential (POCP) is about 50% lower, and the eutrophication potential (EP) is about 70% lower than for 1 kg of citric acid. However, the production location of oxalic acid has a certain influence on its environmental impact (see Table S2 in Supporting Information). Production in China or India would increase the potential of each impact category by ca. 20% mainly driven by a different energy mix available for production. Considering the Ecoinvent data for oxalic acid, the environmental impact of oxalic acid is generally higher than for citric acid. GWP and ADP are about >400% higher, AP is about 30 times higher, and EP is about 10 times higher than for 1 kg of citric acid. As indicated by the negative POCP value, in this case, oxalic acid strongly inhibits ozone formation.

Sodium hypochlorite generally has a lower environmental impact per kg than both citric and oxalic acid considering production only. As already mentioned, the use of sodium hypochlorite is associated with the formation of toxic gases and by-products [11].

The chemical consumption compared with the design can be reduced by 20% for MC and 50% for RC by the optimized design operation mode. This corresponds to a reduction in GWP of about 28% and an annual reduction of 225 tons CO_2eq_ for Henriksdal at design capacity (Table 5). By replacing citric acid with oxalic acid at design consumption, GWP would be reduced by 68% and increased by 217% depending on sustainable and fossil oxalic acid production, respectively. If using the optimized design operation mode and oxalic acid instead of citric, the GWP reduction would be 95% and 1057%, respectively, compared with the design and the two production alternatives.

The total GWP for the design with citric acid and sodium hypochlorite corresponds to an annual impact of 907 tons CO_2eq_ for the future Henriksdal WWTP (Table 5). The pilot trials showed that this could be reduced by 753 tons CO_2eq_ to 155 tons CO_2eq_ per year, corresponding to an 83% reduction in the GWP by using the demand-driven operation mode with sustainably produced oxalic acid. This operational mode was tested for more than one year in the pilot without compromising the membrane performance.

For abiotic depletion and photochemical ozone creation potential, the potential savings are similar at an 82% and 87% reduction, respectively, when the assessment is based on sustainably produced oxalic acid. Using fossil-based oxalic acid, an increase in AD by 20% may take place instead. Using sustainably produced oxalic acid and the demand-driven operation mode, the smallest reduction would be for acidification (45% compared with the design), and the largest saving would be for eutrophication, which could be reduced by 93%. Using fossil-produced oxalic acid, a significant positive effect is calculated for POCP the more fossil oxalic acid is used. This illustrates a dilemma with environmental impact assessments and competing impact categories.

It is interesting to observe that the environmental impact of sustainably produced oxalic acid at design consumption is lower than the impact of the optimized consumption of citric acid for all impact categories.

### 3.4. Costs

With average costs for citric acid, oxalic acid, and sodium hypochlorite of 3600 SEK/ton (12% solution), 15,000 SEK/ton (40% solution), and 3000 SEK/ton (7.2% solution), respectively, the cost for the various operation modes was calculated, as presented in Table 6. The expected cost for cleaning chemicals in the Henriksdal WWTP amounts to ca. 14 M SEK annually as the plant is designed to use sodium hypochlorite and citric acid. Replacing citric acid with oxalic acid would save 20% of cleaning chemicals, not considering other costs related to different handling of oxalic acid. The main savings, however, can be achieved by implementing the optimized or demand-driven cleaning modes. Using oxalic acid in a demand-driven operation mode has the potential to save 75% of the cost of cleaning chemicals, amounting to more than 10 M SEK annually.

Because of the obvious significant savings possible and no reduction in the process performance, the membrane supplier has already agreed to implement at least parts of the optimized cleaning strategy in the full-scale operation of the first treatment line at Henriksdal WWTP. This is currently expected to save 4 M SEK per year for future Henriksdal (with seven treatment lines).

## 4. Conclusions

Long-term pilot trials have demonstrated that municipal wastewater treatment using MBR processes can be achieved with significantly fewer chemicals for membrane cleaning than traditionally required. This finding is crucial, as chemical usage for cleaning is one of the primary disadvantages of MBR processes. The study results, along with the successful implementation of some findings at the full-scale MBR installation at Stockholm’s Henriksdal WWTP, suggest that reducing chemical use in cleaning can substantially enhance the sustainability of both current and future MBR installations.

The novel membrane cleaning strategies investigated in this study indicate considerable potential for operational cost savings and reduced environmental impact due to decreased chemical use. For Stockholm’s Henriksdal WWTP, adopting these alternative cleaning strategies could result in annual savings equivalent to 750 tons of CO_2eq_ emissions and 10.5 million SEK in operational costs, without compromising membrane performance, as evidenced by the long-term pilot trials.

Given the increasing adoption of MBR processes for municipal wastewater treatment to meet stricter emission regulations and promote circular water management, these results contribute significantly to achieving more sustainable wastewater treatment for existing and new MBR installations. Furthermore, this research may enhance the attractiveness and competitiveness of MBR processes compared with conventional treatment systems.

This study also revealed that when comparing citric acid and oxalic acid for membrane cleaning, the use of sustainably produced oxalic acid can further reduce the environmental impact of MBR processes. These results emphasize the importance of considering the entire production process of chemicals used in MBR cleaning to improve sustainability. In addition, oxalic acid avoids the risk of phosphorus release during membrane cleaning, which is critical when meeting stringent phosphorus effluent limits.

Given that the current study focused on specific chemicals and optimization strategies, there is potential for further improvements in chemical use and overall resource efficiency, such as reducing the need for precipitation chemicals and additives. However, during the partial implementation of these strategies at Stockholm Henriksdal WWTP, the importance of considering aspects like the membrane supplier’s warranty, which includes cleaning procedures, became evident. This example of successful implementation at Stockholm Henriksdal WWTP, however, suggests that suppliers are open to adopting new research findings that improve the sustainability of MBR processes if it can be ensured that operational performance is not affected.

One significant potential benefit of reduced chemical cleaning in MBR processes is the expected extension of membrane service life, as membrane aging is primarily driven by chemical degradation. Each additional year of membrane operation can lead to significant cost savings and reduced environmental impacts, as more wastewater can be treated during the membrane’s lifetime.

## Figures and Tables

**Table 1:** Cleaning specification according to the membrane suppliers’ recommendations. Either citric or oxalic acid was used.

	Unit	Sodium Hypochlorite	Citric Acid	Oxalic Acid
Maintenance cleaning (MC)	Frequency	2 per week	1 per week	1 per week
Recovery cleaning (RC)	Frequency	2 per year	2 per year	2 per year
Backpulse flux during MC	L/(m^2^·h)	20	20	20
Backpulse flux during RC	L/(m^2^·h)	34	34	34
Concentration in MC backpulse	mg/L	200	2000	1300
Concentration in RC backpulse	mg/L	1100	2200	1500

**Table 2:** Future effluent requirements and pilot scale operation results (n.a. = not analyzed).

Parameter	Limit	Comment	2016 *	2017	2018	2019	2020	2021
BOD_7_ (mg/L)	5	yearly average	<2	n.a.	n.a.	n.a.	n.a.	n.a.
TN (mg/L)	6	yearly average	4.6	4.8	4.6	4.4	3.9	3.9
NH_4_-N (mg/L)	2	average Apr–Oct	0.2	0.5	0.4	0.6	0.8	0.2
TP (mg/L)	0.20	yearly average	0.33	0.14	0.14 **	0.12	0.05	0.13

* June to December. ** Excluding 16 weeks (w. 26–41) of a special trial without Fe dosage.

**Table 3:** Chemical consumption for membrane cleaning in the pilot for 2020 and the Henriksdal full-scale design.

	Unit	Design Resp. Pilot Value	Full-Scale Fraction
**Chemical consumption RC**			
Citric acid (51%)			
Design mode	L/m^3^	0.00061	100%
Optimized design operation mode	L/m^3^	0.00031	50%
Demand-driven operation mode	L/m^3^	0.00027	44%
Oxalic acid (8%)			
Design mode	L/m^3^	0.0033	100%
Optimized design operation mode	L/m^3^	0.00165	50%
Demand-driven operation mode	L/m^3^	0.00146	44%
Sodium hypochlorite (12%)			
Design mode	L/m^3^	0.0014	100%
Optimized design operation mode	L/m^3^	0.00069	50%
Demand-driven operation mode	L/m^3^	0.00062	44%
**Chemical consumption MC**			
Citric acid (51%)			
Design mode	L/m^3^	0.0020	100%
Optimized design operation mode	L/m^3^	0.0015	80%
Demand-driven operation mode	L/m^3^	0.00091	48%
Oxalic acid (8%)			
Design mode	L/m^3^	0.0100	100%
Optimized design operation mode	L/m^3^	0.0080	80%
Demand-driven operation mode	L/m^3^	0.00304	32%
Sodium hypochlorite (12%)			
Design mode	L/m^3^	0.0018	100%
Optimized design operation mode	L/m^3^	0.0014	80%
Demand-driven operation mode	L/m^3^	0.00039	24%

**Table 4:** Environmental impact of citric acid, oxalic acid, and sodium hypochlorite.

Category (CML2001)	Citric Acid 1 kg, 100%	Oxalic Acid * 1 kg, 100%	Oxalic Acid ** 1 kg, 100%	Sodium Hypochlorite 1 kg, 100%
Global warming potential (GWP 100 years), excl biogenic carbon [kg CO_2eq_]	2.72	1.828	12.4	0.922
Abiotic depletion (ADP fossil) [MJ]	34.7	24.59	181	12.7
Acidification potential (AP) [kg SO_2eq_]	0.0182	0.0091	0.313	0.00263
Eutrophication potential (EP) [kg phosphate eq.]	0.00867	0.00267	0.0691	0.000295
Photochem. ozone creation potential (POCP) [kg ethene eq.]	0.00106	0.000566	−0.0996	0.000159

* Sustainable production process (simplified LCA-model developed), se in methods ** Fossil production process (Ecoinvent data), se in methods.

**Table 5:** Calculated total annual environmental impact of citric acid, oxalic acid, and sodium hypochlorite for the expected amounts used in the full-scale design for Henriksdal based on usage in the pilot.

	Design Mode	Optimized Design Operation Mode	Demand-Driven Operation Mode
**Global Warming Potential [kg CO_2eq_]**			
Citric acid	828,700	604,600	391,900
Oxalic acid (sustainable production)	366,900	266,200	129,000
Oxalic acid (fossil production)	2,488,900	1,805,800	875,200
Sodium hypochlorite	78,670	52,700	25,700
**Abiotic Depletion [MJ]**			
Citric acid	10,572,100	7,713,600	4,999,800
Oxalic acid (sustainable production)	4,935,600	3,581,100	1,735,600
Oxalic acid (fossil production)	36,329,700	26,359,200	12,775,300
Sodium hypochlorite	1,083,600	725,800	354,600
**Acidification Potential (AP) [kg SO_2eq_]**			
Citric acid	5545	4046	2622
Oxalic acid (sustainable production)	1827	1325	642
Oxalic acid (fossil production)	62,800	45,600	22,100
Sodium hypochlorite	224	150	73
**Eutrophication Potential (EP) [kg Phosphate eq.]**			
Citric acid	2641	1927	1249
Oxalic acid (sustainable production)	536	389	188
Oxalic acid (fossil production)	13,900	10,000	4877
Sodium hypochlorite	25	17	8
**Photochem. Ozone Creation Potential (POCP) [kg Ethene eq.]**			
Citric acid	323	236	153
Oxalic acid (sustainable production)	114	82	40
Oxalic acid (fossil production)	−200,000	−145,000	−70,300
Sodium hypochlorite	14	9	4

**Table 6:** Calculated annual costs in SEK for cleaning chemicals for the three operation modes for future Henriksdal.

	Design Mode	Optimized Design Operation Mode	Demand Driven Operation Mode
Citric acid	11,460,000	8,360,000	5,420,000
Oxalic acid	8,360,000	5,460,000	2,650,000
Sodium hypochlorite	2,560,000	1,710,000	840,000
Total cost sodium hypochlorite + citric acid	14,020,000	10,080,000	6,260,000
Total cost sodium hypochlorite + oxalic acid	10,920,000	7,170,000	3,480,000

## Data Availability

The original contributions presented in the study are included in the article and Supplementary Materials, further inquiries can be directed to the corresponding author.

## Supplementary Materials

The following supporting information can be downloaded at: https://www.mdpi.com/article/10.3390/membranes14060126/s1, Table S1: Overview of the trials linked to reduced resource consumption at MC; Table S2: Environmental impact of oxalic acid at the three potential production locations including Spain, China, and India.
