# Supplementary material for: Chemical-Saving Potential for Membrane Bioreactor (MBR) Processes Based on Long-Term Pilot Trials
Source: Membranes (Basel). 2024 May 29;14(6):126. doi: 10.3390/membranes14060126 (PMC11205864; doi:10.3390/membranes14060126)
Supplement: Supplementary file 1 [file membranes-14-00126-s001.zip › membranes-2994223-supplementary.pdf]

Table S1. Overview of trials linked to reduced resource consumption at MC.

Trial	Start	Days	Description
B1	Apr 2016	363	Baseline—Citric acid was used for both MTs. Cleaning followed the design.
B2	Apr 2017	139	Baseline—Citric acid (MT2) compared to oxalic acid (MT1).
T1	Sep 2017	296	Citric acid (MT2) compared to oxalic acid (MT1)—7 backpulses (BP) both MTs.
T2	Jun 2018	19	Recovery period. Brief chemical swap; citric acid to MT1 and oxalic acid to MT2.
T3	Jul 2018	38	Trials with reduced BP count for oxalic acid, standard procedure for citric acid.
T4	Aug 2018	64	Experiment with reduced BP count and 20% longer time between MC with oxalic acid.
T5	Oct 2018	52	No MC with oxalic acid.
T5	Dec 2018	1	One MC with oxalic acid.
T5	Dec 2018	50	No MC with oxalic acid.
T6	Feb 2019	40	Trials with reduced number BP and 100% longer time between MC with oxalic acid.
T7	May 2019	116	Synchronized MC with sodium hypochlorite followed by acid MC. Acid MC on the same night as hypochlorite MC, a few hours later, every 4th sodium hypochlorite MC for MT1 and every other sodium hypochlorite MC for MT2.
T8	Aug 2019	44	Oxalic acid pumping reduced to 80% chemical flow during pumping.
T9	Oct 2019	20	Citric acid MC with reduced chemical amount, oxalic acid same as previous period.
T10	Oct 2019	97	Oxalic acid 80% chemical flow, 7 BPs, every 4th sodium hypochlorite MC.<br>Citric acid 100% chemical flow, 7 BPs, every 4th sodium hypochlorite MC.
T11	Feb 2020	49	Oxalic acid 80% chemical flow, 7 BPs, every 4th sodium hypochlorite MC.<br>Citric acid 100% chemical flow, 9 BPs, every 4th sodium hypochlorite MC.
T12	Mar 2020	77	Sodium hypochlorite replaced with water for MC.
T13	Jun 2020	92	No sodium hypochlorite MC until permeability was below a threshold (<150 lmh/bar). Acid MC performed every other week (at half the standard frequency).
T14	Sep 2020	34	Recovery period.
T15	Oct 2020	420	Demand-based control of hypochlorite MC using fouling control algorithm. Acid MC every 14 days.

Table S2. Environmental impact for oxalic acid at the three potential production locations Spain, China and India.

Impact category.	Spain	China	India
Global Warming Potential (GWP 100 years), excl biogenic carbon [kg $CO_{2\,eq}$]	1.828	2.229	2.376
Abiotic Depletion (ADP fossil) [MJ]	24.59	27.82	29.50
Acidification Potential (AP) [kg $SO_{2\,eq}$]	0.0091	0.0105	0.0190
Eutrophication Potential (EP) [kg Phosphate eq.]	0.00267	0.00279	0.00306
Photochem. Ozone Creation Potential (POCP) [kg Ethene eq.]	0.000566	0.000769	0.001009
